# Predisposing factors of using cosmetics in Iranian female students: application of prototype willingness model

**DOI:** 10.3389/fpsyg.2024.1381747

**Published:** 2024-06-13

**Authors:** Shandiz Moslehi, Asghar Tavan, Sajjad Narimani, Fatemeh Ahmadi, Masoomeh Kazemzadeh, Nadia Sedri

**Affiliations:** ^1^Health Management and Economics Research Center, Health Management Research Institute, Iran University of Medical Sciences, Tehran, Iran; ^2^Department of Health in Disasters and Emergencies, School of Health Management and Information Sciences, Iran University of Medical Sciences, Tehran, Iran; ^3^Health in Disasters and Emergencies Research Center, Institute for Futures Studies in Health, Kerman University of Medical Sciences, Kerman, Iran; ^4^Department of Health in Disasters and Emergencies, School of Health Management and Information Sciences, Iran University of Medical Sciences, Tehran, Iran; ^5^Department of Nursing and Midwifery, School of Nursing, Social Determinants of Health Research Center, Ardabil University of Medical Sciences, Ardabil, Iran; ^6^Students Research Committee, School of Germi Nursing, Ardabil University of Medical Sciences, Ardabil, Iran; ^7^Nursing Research Center, Kerman University of Medical Sciences, Kerman, Iran

**Keywords:** attitude, cosmetics, prototype willingness model, students, subjective norms

## Abstract

**Background and aim:**

The use of cosmetics among Iranian teenagers and youths has increased more than ever before. This study investigated the predisposing factors of cosmetic use in female students of Ardabil University of Medical Sciences by using the prototype willingness model (PWM).

**Methods:**

This cross-sectional study was conducted with 384 students, selected based on multistage sampling. Data were collected using a two-part questionnaire that included demographic variables and PWM questions. Then multiple regression analysis was used in SPSS (version 20).

**Results:**

There was a significant difference in the frequency of daily cosmetic use based on the education levels (*F* = 3.845, *p*-value = 0.034). The average daily use of cosmetics was higher in students whose use of cosmetics was high in their family (*p* = 0.024) and friends (*p*-value = 0.023). Prototypes were the strongest predictor of using cosmetics (OR = 1.317, *p*-value <0.001), followed by attitude (OR = 1.241, *p*-value <0.001).

**Conclusion:**

Prototypes (social imagination) and attitudes were the main predictors of using cosmetics among female students. To be effective in targeting cosmetic use, interventions must target both social and individual paths.

## Background

College students, entering education and living independently from their parents, experience a new environment on campus that can potentially affect their health behaviors (Narimani et al., 2020). One of the most important health behaviors is the use of cosmetics. Despite the health risks that scientific sources report on the side effects of the use of cosmetics (Panico et al., 2019), the use of cosmetics among teenagers and young adults is increasing today (Ngah et al., 2021). The Federal Food, Drug, and Cosmetic Act (FD&C Act) defines cosmetics as “articles intended to be rubbed, poured, sprinkled, or sprayed on, introduced into, or otherwise applied to the human body for cleansing, beautifying, promoting attractiveness, or altering the appearance” (COSMETIC, 2008).

Iran is one of the main countries that consume cosmetic products in the Middle East around the world, with a financial turnover of more than 2 billion dollars (Ziarati et al., 2012; Ghaderpoori et al., 2020). However, the prevalence of using cosmetics, especially among female students in Iran, has not been investigated sufficiently. In one of the few studies conducted, Hosseini et al. (2014) in western Iran showed that approximately 62 and 50% of students always use lipstick and eyeliner, respectively. Another study in the center of Iran, while confirming the high prevalence of use of cosmetics in women aged 20–29, indicated that among cosmetics, lipstick was the most widely used by women (Dehghani et al., 2017). Although Tarrahi et al. (2016) did not report the frequency of cosmetics use, they showed that the most common type of makeup among female students at Urmia University of Medical Sciences in Iran was traditional makeup, and the least common type of makeup was feminist style. According to the literature review, it seems that investigating the frequency of cosmetic use in Iran is a research necessity.

The reasons for the tendency toward cosmetic products are different among different ethnicities and races. Some of these reasons include achieving the highest level of beauty, having high social prestige, having an attractive presence in celebrations and ceremonies, attracting the attention of the opposite sex, reviving youthful beauty, and increasing the quality of life (Faria-Silva et al., 2020). The competition to establish a correct social relationship and seek superiority through having a beautiful face is one of the reasons why girls and young women use cosmetics (Halla et al., 2018). As much as European girls and women with natural faces desire supremacy, girls and young women in Middle Eastern countries, particularly Iran, seek it through cosmetics and the creation of artificial beauty (Marie et al., 2022). While cosmetics are mostly produced in European countries, their use is high among our female population (Ficheux et al., 2019).

The results of a qualitative study in Iran show that young women’s makeup practices are largely related to their full immersion in the experimental and creative aspects of makeup use and ways to uplift their tired spirits (Jafari and Maclaran, 2014). Another study in Iran showed higher socio-economic status, frequent use of social media, lower health literacy, and the desire to differentiate were the main factors in the consumption of cosmetics (Abdolalizadeh et al., 2023). Other studies in Iran have emphasized that body management and facial beauty are the most important motivations for using cosmetic products among student girls (Movahed et al., 2009; Tarrahi et al., 2016).

It seems that exploratory studies regarding the psychological and social aspects of using cosmetics are insufficient, and these studies should be prioritized. In health education, there are various models for predicting health behavior, one of which is the prototype willingness model (PWM) (Gibbons et al., 2020). According to PWM, there are two main pathways for behavioral preference: the first is the reasoned action path, which includes analytical and argumentative processes with the structures of attitude, subjective norms, and behavioral intentions. Another path is social reactions, which indicate the impact of prototypes on behavior through behavioral willingness (Schreurs et al., 2020).

Frequent use of cosmetics may increase the absorption of heavy metals when eating lipstick or sweating on a skin surface covered with cream or other cosmetics (Ghaderpoori et al., 2020). While the skin should play a protective role against the entry of harmful substances, cosmetic products that are used in sensitive areas such as the lips, around the eyes, or the skin of the face expose the consumer to more metals (Arshad et al., 2020). Cosmetics contain ingredients that have been linked to many diseases, including cancer, birth defects, reproductive problems, and developmental problems (Hadi et al., 2020).

A systematic review of studies on the measurement of heavy metals in cosmetics in Iran showed that the margin of safety (MoS) values calculated for various metals were higher than the safe standard set by the World Health Organization (WHO) (Ghaderpoori et al., 2020). Nevertheless, a survey has revealed that Iranian women have a negative attitude and lack of awareness about the dangers of overusing cosmetics.

This is despite the fact that risk perception is an important issue in public health (Tarrahi et al., 2016), especially in the issue of using cosmetics (Faria-Silva et al., 2020), which emphasizes identifying the determinants of health behavior (Rezaei et al., 2022).

Considering the possible risks of excessive use of cosmetics and the need to determine the effective factors, this study was conducted with the aim of investigating the frequency and predisposing factors of cosmetics use among female students of Ardabil University of Medical Sciences based on PWM.

## Methods

### Study design and participants

This online survey was conducted with 384 female students of Ardabil University of Medical Sciences, in northwestern part of Iran. Samples were selected through social media (WhatsApp and Telegram). To determine the sample size, the literature review lacked similar studies. However, in a pilot study that was conducted to determine the reliability of the questionnaire, the results indicated that 55% of students use cosmetics three or more times a day. Considering *p* = 0.55 and d = 0.05, the sample size was estimated to be 380 people. During the 3 weeks that the questionnaire was available online to the respondents, 510 people visited and 384 students completed the questionnaire. The inclusion criteria were female gender, consent to participate in the study, and studying at Ardabil University of Medical Sciences. Subjects were excluded if their questionnaires were incomplete.

### Measurements

A researcher-made questionnaire in two parts was used to collect data. The first part was a demographic and contextual checklist about age, education grade, parents’ education, and family, cosmetic use in the family and friends, age of first cosmetic use, place for cosmetics, and cosmetic use after waking up. The second part included 42 questions on constructs of PWM, which had six sub-scales. The structure of the PWM questionnaire was adapted, so the results of other studies that evaluated health behaviors in Iran based on PWM were used (Rahimi and Javadi, 2018; Mirzaie Alavijeh et al., 2019; Bashirian et al., 2020; Manoochehri et al., 2021).

In addition, the content of the questions was determined by reviewing the texts and using the opinions of the research team. The way to evaluate the validity and reliability of the questionnaire is presented in the following.

The first sub-scale was cosmetic behavior, which examined the daily use of cosmetics per day with a four-choice question (one time, two times, three times, and four times or more). The second sub-scale was behavioral intention, which had four questions such as “I plan to apply makeup every time I go out” with a 5-point Likert scale ranging from 1 (strongly disagree) to 5 (strongly agree). The third sub-scale was behavioral willingness, which had eight questions such as “seeing the make-up of celebrities and their economic success makes me do make-up” with a 5-point Likert scale ranging from 1 (strongly disagree) to 5 (strongly agree). The fourth sub-scale was the prototypes (social imagination), which had 15 questions with a 5-point Likert scale ranging from 1 (completely inappropriate) to 5 (completely appropriate). Prototypes show personal positive and negative judgments, such as beautiful, social, brave, lovely, showy, obsessive, cute, and unfaithful, about people who wear makeup. The fifth sub-scale was subjective norms, with five questions ranging from 1 (strongly disagree) to 5 (strongly agree), such as “my friends’ opinions about makeup are important to me” or “the opinion of the faculty officials about makeup is important to me” and “my parents’ opinion about makeup is important to me.” The sixth sub-scale measured attitude and included nine questions ranging from 1 (strongly disagree) to 5 (strongly agree), such as “if I do makeup, I look more beautiful” or “makeup relaxes me” and “Makeup makes me look cute.”

The content validity of the questionnaire was evaluated through a panel of experts consisting of six health education and health promotion experts and two epidemiologists. The values obtained for content validity index (CVI) and content validity ratio (CVR) were 0.86 and 0.92, respectively. The reliability of the questionnaire was confirmed by Cronbach’s alpha of 0.81 in a preliminary study on 40 students. Furthermore, Cronbach’s alpha coefficient for different subscales of PWM was: behavioral intention (0.763), behavioral willingness (0.715), prototypes (0.97), subjective norms (0.822), and attitude (0.81).

### Ethical approval

Since data collection was online, in order to comply with ethical principles, an informed consent form was presented on the first page of the online questionnaire. On the mentioned page, the participants were assured that all their information will be confidential and anonymous, and the participants indicated their agreement to participate in the study by checking a box. Ethic approval was obtained from the ethics committee of Ardabil University of Medical Sciences (IR.ARUMS.REC.1399.308).

### Statistical analysis

The data were entered in SPSS software version 20. The data normality was confirmed by the Kolmogorov–Smirnov test. Mean values with the standard deviation were used for descriptive statistics. *T*-test and one-way analysis of variance (ANOVA) were used to compare the average use of cosmetics in the categories of demographic and contextual variables. Pearson’s correlation test was used to check the correlation between the constructs of the PWM. Finally, a logistic regression test was performed to determine the predictors of cosmetic use. Binary logistic regression assumes that the dependent variable must have two states. Therefore, according to the median of 3 for cosmetics use, we classified the people who used cosmetics less than three times a day in the low consumption group and the people who used three or more times in the high consumption group. A significance level of *p* < 0.05 was adopted in this study.

## Results

The mean age of the participants in this study was 23.11 ± 2.52 years. The average daily use of cosmetics in the participants was 3.18 ± 1.49. Furthermore, the median value of 3.00 indicated that at a minimum of 50% of people wear makeup three times a day. More than half of the people were bachelor students (200 people, 52.8%). The majority of students reported that the use of cosmetics is high in their family (250 people, 75%) and in their friends (287 people, 87%). The highest rate of cosmetics use was during video calls on social media (34.9%). Most of the students stated that their first daily makeup happens within half an hour after waking up (78.3%). More details about demographic information are provided in Table 1.

**Table 1:** Demographic characteristics and comparison of average use of cosmetics.

Variables	Categories	*N* (%)	Daily cosmetic use	*p*-value
Education level	Bachelor	200 (52.1)	2.92 ± 1.34	*F* = 3.845 ***p*-value** **< 0.034** Doc > Msc, Bachelor
	MSc	14 (3.6)	2.1 ± 1.41	
	Doctorate	170 (44.3)	3.60 ± 1.58	
Mother’s occupation	Housewife	138 (35.9)	3.44 ± 1.70	*F* = 0.579 *p*-value <0.630
	Employee	150 (39.1)	3.1 ± 1.32	
	Retired	49 (12.8)	2.92 ± 1.32	
	Other	47 (12.2)	3.1 ± 1.59	
Father’s occupation	Employee	150 (39.1)	3.01 ± 1.63	*F* = 0.559 *p*-value <0.634
	Private sector	115 (29.9)	3.29 ± 1.36	
	Retired	54 (14.1)	2.86 ± 1.41	
	Other	65 (16.9)	3.50 ± 1.50	
Cosmetic use in the family	Low level	100 (26)	2.60 ± 1.26	*t* = 2.290 ***p*-value = 0.024**
	High level	284 (74)	3.38 ± 1.52	
Cosmetic use in the friends	Low level	50 (13)	2.31 ± 1.25	*t* = 2.308 ***p*-value = 0.023**
	High level	334 (87)	3.31 ± 1.49	
Age of first cosmetic use	Under 12 years old	80 (20.8)	3.40 ± 1.32	*F* = 0.725 *p*-value <0.432
	12–18 years old	185 (48.2)	3.18 ± 1.43	
	Upper 18 years old	119 (31.0)	3.0 ± 1.38	
The most used place for cosmetics	Home	40 (10.4)	2.22 ± 1.12	*F* = 0.832 *p*-value <0.125
	Social media chatting	134 (34.9)	3.42 ± 1.62	
	Party	120 (31.3)	3.12 ± 1.28	
	University	90 (23.4)	3.32 ± 1.69	
Cosmetic use after waking up	Up to 10 min	181 (47.1)	3.30 ± 1.32	*F* = 0.875 *p*-value <0.254
	10–30 min	120 (31.2)	3.1 ± 1.44	
	More than half an hour	84 (21.7)	2.95 ± 1.26	

There was a significant difference in the frequency of daily cosmetics use based on the education levels, and Tukey’s post-hoc test confirmed that it was higher in general doctoral students than the other two groups (*F* = 3.845, *p*-value = 0.034). The average daily use of cosmetics was higher in students whose use of cosmetics was high in their family (*p* = 0.024) and their friends (*p*-value = 0.023). Other results related to the comparison of average daily use of cosmetics according to demographic and contextual variables are presented in Table 1.

Pearson’s correlation test determined that all constructs of PWM are directly correlated with cosmetic use. Among the constructs, prototypes (*r* = 0.58, *p* < 0.001) had a stronger correlation with cosmetics use, followed by attitude (*r* = 0.56, *p* < 0.001). Other correlations are shown in Table 2.

**Table 2:** Correlation matrix of the constructs of the prototype willingness model.

Variables	Attitude	Subjective norms	Behavioral willingness	Behavioral intention	Prototypes	Cosmetic use
Attitude	1					
Subjective norms	0.53**	1				
Behavioral willingness	0.49**	0.45**	1			
Behavioral intention	0.42**	0.51**	0.44**	1		
Prototypes	0.51**	0.56**	0.42**	0.61**	1	
Cosmetic use	0.56**	0.47**	0.51**	0.53**	0.58**	1

Correlation is significant at the 0.01 level **.

The results of logistic regression (presented in Table 3) showed that PWM constructs had a significant role in predicting the use of cosmetics. Prototypes were the strongest predictor of using cosmetics (OR = 1.317, *p*-value <0.001), followed by attitude (OR = 1.293, *p*-value <0.001). These results show that for each unit of increase in prototypes and attitude, the chance of using cosmetics increases by approximately 32 and 29%, respectively. Other significant structures, in order of predictive power, were behavioral intention (OR = 1.195, *p*-value = 0.024), behavioral willingness (OR = 1.152, *p*-value = 0.028), and subjective norms (OR = 1.142, *p*-value = 0.035). Furthermore, Cox & Snell R Square and Nagelkerke R Square determination coefficients were equal to 0.267 and 0.387, respectively. These results show that approximately 27 to 39% of the probability of using cosmetics three times a day or more is explained by the constructs of the PWM model.

**Table 3:** Logistic regression analysis of prototype willingness model constructs for cosmetic use.

Constructs of prototype willingness model	B	SE	Odd Ratio (95.0% C.I. for OR)	*p*-value
Attitude	0.241	0.04	**1.293** (1.241–1.352)	**0.001**
Subjective norms	0.163	0.05	**1.142** (1.075–1.211)	**0.035**
Behavioral intention	0.192	0.09	**1.195** (1.125–1.269)	**0.024**
Prototypes	0.261	0.05	**1.317** (1.265–1.372)	**0.001**
Behavioral willingness	0.187	0.12	**1.152** (1.102–1.208)	**0.028**

Dependent variable: cosmetic use behavior Cox & Snell R Square = 0.267, Nagelkerke R Square = 0.387.

## Discussion

The present research was carried out to determine the factors affecting cosmetics use among female students at Ardabil University of Medical Sciences using a prototype willing model. The results showed that all students used cosmetics, and at least 50% of people wear makeup three times a day. These findings are consistent with the study of Husain (2019) in Saudi Arabia, who showed that more than 93% of students used cosmetics daily. In another study in Sri Lanka, almost the entire study population (96.4%) used one or more cosmetic or personal care products. Of course, less than in the current study, the use of cosmetics for face makeup was approximately 65% (Udayanga et al., 2024). Another study in Turkey showed that approximately 62% of students use cosmetics daily, which was less than the present study (Olcer et al., 2023). A study on Egyptian students also indicated the overall prevalence of the use of cosmetic products at 87.5% (El Emam Hafez El Emam et al., 2022). Presenting these rates is important because it shows that the use of cosmetics for face makeup among the students in the present study is higher than in other studies, and it points out the need for studies to determine the effective factors.

The results showed that the use of cosmetics by students was related to higher education and the high use of cosmetics by family and friends. Students of general medicine, pharmacy, and dentistry used cosmetics more frequently than Bachelor and Master of Science students. A study in Brazil found that higher education is associated with greater use and more time spent using cosmetics (Mitterer-Daltoé et al., 2023). Other studies in the United States and Ethiopia have indicated the effect of higher education on greater use of cosmetics (Chukwuma, 2018; Getachew and Tewelde, 2018). It seems that the higher social prestige of medical, pharmaceutical, and dental fields of study in Iran leads the students of these fields to be beautiful and use more cosmetics. Perhaps the social prestige and higher self-confidence among these students can be mentioned as the mediating factors, something that has been confirmed in other studies (Bottesi et al., 2018; Mobil et al., 2019).

The results of several studies in the world have shown that many cosmetics contain substances harmful to people’s health, including heavy metal impurities, microbial contamination, preservatives, and other health concerns (Lavilla et al., 2018; Panico et al., 2019; Akhand et al., 2023).

Therefore, it is very important to gain a correct understanding of the factors affecting the tendencies or behaviors toward using cosmetics in different groups of society, especially female students. In this study, we used a two-headed model to investigate cosmetics use behavior, PWM, with two paths of reasoned action and social reaction. The first pathway, which is reasoned action, involves behavioral intention (influenced by attitude and subjective norms) as a proximal antecedent to behavior, and the second pathway, which involves social reaction, involves behavioral intention (influenced by prototype) as an additional proximal antecedent (Gibbons et al., 2020).

The logistic regression analysis showed that all the constructs of the PWM had a significant role in predicting the behavior of cosmetics. Prototype (social imagination) was the strongest predictor of cosmetic use behavior. The powerful role of the prototype in this study shows the influence of social mentality and modeling on the behavior of using cosmetics. In a multi-ethnic study in Australia, Wagstaff found that social factors did not influence the weekly frequency of cosmetic use in women, contrary to sociological arguments that cosmetic use is driven by social pressures. The results showed that mating strategies and narcissism were the best predictors of cosmetic use (Wagstaff, 2018).

On the contrary, reviewing past studies, Jones and Kramer (2016) concluded that cosmetic use is effective in improving social perceptions because it makes people appear healthier, earn more money, and appear more likable, trustworthy, and authoritative. In one interesting study, people rated faces with makeup (either light or heavy) as more attractive than faces without makeup. In addition, faces with heavy makeup were rated significantly higher in terms of attractiveness and socio-sexuality than faces with light makeup (Aguinaldo and Peissig, 2021). The mentioned study showed that people choose heavy and light makeup faces as acceptable social examples.

Prototypes are subjective examples of social comparison. In this comparison, if imaginary or real social examples and models are placed on the upper hand and the individual is on the lower hand, the desire to fix this gap increases (Abdel Hamid El Khoreiby, 2021). Usually, women who are dissatisfied with their body image are more prone to social comparison and make this comparison with imaginary models or attractive media models (Davis, 2013). This is exemplified in beauty-seeking behavior, where a person considers a prototype that is beautiful, bold, and attractive with makeup. All these items create a behavioral willingness to makeup in a person, which can lead to the use of cosmetics.

We explained the social path through prototypes, but individual beliefs and preferences start on the path of rational action and attitudes. The most important attitude component for using cosmetics is to be attractive and be seen as attractive. Reviewing the literature, Robertson and Kingsley (2021) showed that attractiveness brings many benefits in the areas of relationships with the opposite sex, winning lawsuits, economic success, employment and career promotion, and educational opportunities. This is the reason why people use cosmetics to gain the advantage of attractiveness. Pourrajabi and Ghobadi in Iran also found that attitudes such as becoming beautiful, attractive, and looking cleaner with the use of cosmetics have made makeup popular among women (Pourrajabi and Ghobadi, 2020). A qualitative study showed that individual attitudes were the most important factor in African American women’s use of cosmetics as a magical enhancer of their features. All women expressed positive attitudes about using cosmetics, and many believed that cosmetics magically made them more beautiful, bolder, and more attractive (Davis, 2013).

A positive attitude toward makeup and receiving a good feeling in psychological aspects such as a feeling of relaxation, success, self-confidence, and creativity are among the factors that can lead a person to use cosmetics. If we can consider prototypes or social imagination as a woman’s view of others who wear makeup, we can consider attitudes as a person’s view of herself who wears makeup. The results of a study in South Africa showed that female consumers of cosmetics had a favorable attitude toward beauty products, which was influenced by their subjective norms. As subjective norms, group influence, including the opinions of friends, family, and peers, and media influence predicted people’s attitudes (Dalziel and De Klerk, 2021). Robertson et al. found that the opinions of classmates and friends about the use of cosmetics are important and encourage people to wear makeup.

As we discussed, factors in the paths of logical action and social reaction were effective in the use of cosmetics by girls. In other studies, these factors have been discussed with other terms, but what is important is the significant role of individual and social components in the behavior of cosmetics.

Limitations: Our study was conducted among female students of the University of Medical Sciences, and the answers were obtained based on a self-report questionnaire. Considering the increase in the use of cosmetics among young boys and teenagers, it is recommended that further studies be conducted among all young people, both boys and girls. Furthermore, along with cross-sectional studies and questionnaires, it is possible to enrich the findings by conducting qualitative studies through interviews.

## Conclusion

Prototypes (social imagination) and attitudes (as an internal individual factor) were the main predictors of using cosmetics among female students. Furthermore, subjective norms, behavioral intention, and behavioral willingness were effective factors in using cosmetics. The sum of these results shows that individual and social factors, in a complex and continuous interaction, lead girls to use cosmetics more and more, and what seems to be neglected are the risks of using these substances. Therefore, although more research is needed to investigate people’s motivations toward cosmetics, it is suggested that educational interventions reduce the frequency of cosmetics using reasoned action and social reaction paths for female students. In these interventions, prototypes that use lighter, less harmful, and healthier makeup can be used as correct behavioral models in the implementation of training.

## Data availability statement

The original contributions presented in the study are included in the article/supplementary materials, further inquiries can be directed to the corresponding author.

## Ethics statement

The studies involving humans were approved by Ethic approval was obtained from the ethics committee of Ardabil University of Medical Sciences (IR.ARUMS.REC.1399.308). The studies were conducted in accordance with the local legislation and institutional requirements. Written informed consent for participation in this study was provided by the participants’ legal guardians/next of kin.

## Author contributions

SM: Conceptualization, Investigation, Supervision, Validation, Writing – review & editing. AT: Formal analysis, Methodology, Resources, Writing – review & editing. SN: Conceptualization, Investigation, Methodology, Resources, Validation, Visualization, Writing – original draft, Writing – review & editing. FA: Data curation, Writing – original draft. MK: Writing – original draft. NS: Validation, Visualization, Writing – review & editing.
